# Sex Differences in Spatial Memory in Brown-Headed Cowbirds: Males Outperform Females on a Touchscreen Task

**DOI:** 10.1371/journal.pone.0128302

**Published:** 2015-06-17

**Authors:** Mélanie F. Guigueno, Scott A. MacDougall-Shackleton, David F. Sherry

**Affiliations:** 1 Department of Biology, University of Western Ontario, London, Ontario, Canada; 2 Department of Psychology, University of Western Ontario, London, Ontario, Canada; 3 Advanced Facility for Avian Research, University of Western Ontario, London, Ontario, Canada; University of Lethbridge, CANADA

## Abstract

Spatial cognition in females and males can differ in species in which there are sex-specific patterns in the use of space. Brown-headed cowbirds are brood parasites that show a reversal of sex-typical space use often seen in mammals. Female cowbirds, search for, revisit and parasitize hosts nests, have a larger hippocampus than males and have better memory than males for a rewarded location in an open spatial environment. In the current study, we tested female and male cowbirds in breeding and non-breeding conditions on a touchscreen delayed-match-to-sample task using both spatial and colour stimuli. Our goal was to determine whether sex differences in spatial memory in cowbirds generalizes to all spatial tasks or is task-dependant. Both sexes performed better on the spatial than on the colour touchscreen task. On the spatial task, breeding males outperformed breeding females. On the colour task, females and males did not differ, but females performed better in breeding condition than in non-breeding condition. Although female cowbirds were observed to outperform males on a previous larger-scale spatial task, males performed better than females on a task testing spatial memory in the cowbirds’ immediate visual field. Spatial abilities in cowbirds can favour males or females depending on the type of spatial task, as has been observed in mammals, including humans.

## Introduction

Some animals show adaptive specialization of spatial ability [1, 2, 3]. Food-storing birds remember the locations of large numbers of scattered food caches. Spatial memory differs between food-storing and non-storing species [4, 5] and between populations of the same food-storing species that differ in their reliance on stored food [6]. Adaptive specialization can also lead to sex differences in spatial ability. Polygynous male meadow voles (*Microtus pennsylvanicus*) have larger home ranges, better spatial memory, and a larger hippocampus than females, sex differences that are not found in monogamous species of *Microtus* where females and males have similar home ranges [7, 8, 9, 10, 11]. Similar sex differences in spatial memory occur in polygynous deer mice (*Peromyscus*) [12, 13] and laboratory mice [14].

Sex differences in spatial ability are not, however, simply a matter of better or poorer performance by one sex or the other. Female and male laboratory rats and mice use different dissociable kinds of spatial information for orientation and navigation [14, 15]. Male mice and rats rely predominantly on geometric information (i.e., distant landmarks), whereas females use predominantly feature information (i.e., local landmarks) [14, 15, 16]. Consistent with this sex difference in the use of spatial information, male mice, rats, and humans outperform females on spatial tasks requiring navigation through space or the visualization of three-dimensional or directional information, whereas females outperform males on smaller scale spatial tasks relating to object location [14, 15, 16, 17, 18]. Sex differences in the kind of information used for orientation appear to result from organizational effects of gonadal steroids during development [19].

Sex differences in spatial ability also occur in birds. Male hummingbirds (*Sephanoides sephanoides*) are better than females at remembering the location of high quality nectar sources [20] and female brood parasitic brown-headed cowbirds (*Molothrus ater*) are better than males at remembering the location of a previously baited food source [21]. Astié, Kacelnik, and Reboreda [22] found, in contrast, that female shiny cowbirds (*Molothrus bonariensis*) performed better than males when a colour cue indicated the location of food, but not when spatial location alone was associated with food. Sex differences in the use of spatial information, however, do not always occur in birds. Females of three species of hummingbirds (*Selasphorus rufus*, *Hylocharis leucotis*, *Eugenes fulgens)* have the same preference as males for spatial cues over featural cues [23] and the same is true of great tits (*Parus major*) [24].

Some birds use spatial information differently depending on context. Noisy miners (*Manorina melanocephala*) revisit sites where they have *not* previously found food more often when searching for invertebrates than when searching for nectar [25]. European greenfinches (*Carduelis chloris*) predominantly use colour cues to relocate a food site they have encountered only once previously, but switch to spatial cues after repeated encounters with food at the same site [26].

Brown-headed cowbirds are obligate brood parasites and spatial ability likely plays a major role in the reproductive success of females. To successfully reproduce, females must find host nests and then re-visit these nests to assess the stage of completion of the host clutch, to lay their own eggs, and to remove host eggs [27, 28, 29, 30, 31]. Male brown-headed cowbirds do not search for or visit host nests. Females might be expected to perform better than males on spatial memory tasks and indeed, female brown-headed cowbirds outperform males when searching for food hidden in baited cups within a large room [21]. The hippocampus is larger in female than in male brown-headed cowbirds [32] and shiny cowbirds, another brood-parasitic species in which only females search for host nests [33]. In screaming cowbirds (*Molothrus rufoaxillaris*), a related species of cowbird in which both sexes search for nests, females and males have a similar-sized hippocampus [33]. The sex difference in favour of females in both navigational spatial ability and relative size of the hippocampus found in brown-headed cowbirds is thus the reverse of that usually found in mammals.

Female cowbirds only search for host nests during the breeding season. Changes in breeding condition influence hormone levels in both female and male brown-headed cowbirds [21] and both testosterone and estradiol can influence acquisition and performance of spatial tasks [34, 35, 36].

In the current study, we used operant conditioning to compare performance of female and male brown-headed cowbirds on a delayed-matching-to-sample (DMTS) touchscreen task. This task, unlike the task used by [21], does not involve movement though a spatial environment but instead memory for a location in the immediate visual field. We investigated whether female cowbirds perform better than males in general or if superior female performance is associated only with tasks that resemble females’ search for nests. We also compared performance between breeding and non-breeding conditions, inducing breeding condition by manipulation of photoperiod. Finally, we compared memory for both spatial and colour cues on the touchscreen.

We predicted that if female cowbirds have better spatial ability than males in general, then females would perform better than males on the spatial but not on the colour DMTS touchscreen task. In contrast, spatial ability may depend on the nature of the task, as in rodents and humans. Because female cowbirds outperformed males on a previous spatial task that required movement through space [21], we predicted that if sex differences in cowbirds is task-dependent, performance on the spatial touchscreen task would either be similar between the sexes or may favour males.

## Materials and Methods

### Ethics statement

All work was carried out in strict accordance with the recommendations for behavioural research of the Canadian Council on Animal Care. The protocol was approved by The Animal Use Subcommittee of the University Council on Animal Care, Western University, under Animal Use Protocol 2007–001 to DF Sherry. All birds were housed in individual cages with social contact with immediate neighbors on the left and right and with other birds in a group colony room. Birds were provided with enrichment in the form of soft flexible perches as well as solid perches of varying diameters and ping pong balls as toys. Diet was carefully prepared not only to be nutritionally complete but also to provide variety in the form of vitamin supplements and live mealworms. Birds remained in captivity after the study with the same level of care and enrichment.

### Subjects

We captured eight female and eight male cowbirds in April 2011 at the Queen’s University Biological Station near Elgin, Ontario, Canada using the same traps described in [37]. Birds were at least a year old at the time of capture. After capture, birds were transported to the University of Western Ontario where they were housed indoors in individual cages and fed them *ad libitum* for two months until the beginning of training. During this two-month period, birds reached their captive free-feeding weight. Food consisted of a mix of seeds (50% Living World Premium seed mix for budgies: 50% white millet), fruits, vegetables, a modified Bronx Zoo diet for omnivorous birds (eggs, carrots, molasses, brown rice, wheat germ, dog food, exotic gamebird starter, and turkey starter) and oyster shells.

### Touchscreen apparatus, training and software

During training and testing for the touchscreen tasks, we food-restricted subjects to maintain them at 85% of their free-feeding weight, which was similar to their weight at capture from the wild. First, we trained naïve subjects to feed from a food hopper. We then manually shaped them to peck a shape on the touchscreen to access the food hopper. Finally, we trained subjects to peck progressively longer sequences of shapes until the full sequence for the task was reached. See S1 Text for more details.

Touchscreen chambers were 31 cm deep, 36 cm wide, and 34 cm high, and were housed in sound-attenuating booths (Eckel Noise Control Technologies, Morrisburg ON). Subjects pecked a computer monitor that made up one side of the chamber, and was surrounded by a CarrollTouch infrared touchscreen frame (Elo Touch Solutions, Rochester NY). Each monitor and touchscreen frame was connected to a computer that presented stimuli and recorded responses using Experimentor Software, a program developed for this study [38]. See S1 Text for further details.

### Breeding condition manipulations and measurements

#### Photoperiod manipulation

Subjects were tested in non-breeding and breeding conditions (Table 1). Subjects were exposed to varying photoperiods at different points in the study to induce photorefractory and photosensitive states and photostimulated to induce breeding condition [39]. Birds were not tested during the 60 d period when they were held on a short photophase (8 h L: 16 D) to induce photosensitivity between non-breeding and breeding conditions. To ensure that performance was not affected by the order of testing of non-breeding and breeding conditions, birds repeated the entire sequence of training to criterion followed by Progressive Retention Interval (RI) training before Randomized RI testing began for both spatial and colour tasks (Table 1, S1 Text).

**Table 1:** Sequence of testing conditions for all birds.

NON-BREEDING	
Spatial DMTS	Training to criterion
	Progressive retention intervals: 15 sessions
	Randomized retention intervals: 11 sessions + 3 testing sessions
Colour DMTS	Training to criterion
	Progressive retention intervals: 15 sessions
	Randomized retention intervals: 11 sessions + 3 testing sessions
BREEDING	
Spatial DMTS	Training to criterion
	Progressive retention intervals: 15 sessions
	Randomized retention intervals: 11 sessions + 3 testing sessions
Colour DMTS	Training to criterion
	Progressive retention intervals: 15 sessions
	Randomized retention intervals: 11 sessions + 3 testing sessions

Spatial and colour delayed-matching-to-sample (DMTS) tasks were performed in non-breeding condition and breeding condition at varying retention intervals.

In addition to blood sampling (details below), we measured changes in frequency of singing and gonad size to confirm breeding condition (S1 Text).

#### Blood sampling

Touchscreen testing did not occur on blood sampling days, and subjects were not food deprived. For each breeding condition, two blood samples were taken during spatial testing and two more during colour testing, for a total of four samples from each subject per condition. Blood samples were collected within 30 min of entering the housing room. We punctured the brachial vein with a 26-gauge needle and collected approximately 300 μL of blood into heparinized capillary tubes. Blood was kept cool and then centrifuged at 13 g for 10 min within 5 h of collection. The plasma was frozen in glass vials and stored in a freezer until hormone assay. For both the breeding condition and non-breeding condition, two samples taken during spatial testing were pooled, as were the two samples taken during colour testing, for a total of two large samples per subject per breeding/non-breeding condition.

#### Androgen assay

Androgen concentration was assayed using a testosterone enzyme immunoassay (EIA; Cat. #1–2402, Salimetrics). Because the antibody in this kit cross-reacts with dihydrotestosterone and other androgens, we refer to measures as androgen levels. This kit has been validated in songbirds [40]. We diluted plasma samples five times and followed [41] to validate the assay for cowbirds. We assayed a serial dilution of cowbird plasma and compared it to the standard curve using ANCOVA. A non-significant interaction term (F_1,9_ = 0.42, *p* = 0.54) indicated the slopes were similar (R^2^ = 0.84) and that the assay was suitable for cowbirds. Inter-plate variation, based on a pooled cowbird plasma sample and low and high controls, was 9.41%. Intra-assay variation, based on variation between duplicates, was 9.43%. The sensitivity of our assay was 1 pg/mL (two standard deviations from the average value of zero on our four standard curves). Samples below 1 pg/mL were assigned a value of 0.5 pg/mL for statistical analyses.

### Delayed-matching-to-sample (DMTS) tasks

Once the subjects had learned to use the food hopper and touchscreen, we tested them first with a spatial DMTS task, then a colour DMTS task in non-breeding and breeding conditions (Table 1). A trial began by a subject pecking a fixation point located in the middle of the screen to ensure the subject was attending to the screen when testing began (S1 Fig). The fixation point disappeared once pecked and a sample square was presented. White squares with black outlines were presented for the spatial task, and coloured squares for the colour task (S1 Fig, S1 Table). The sample square disappeared when pecked and an RI followed during which a blank white screen appeared. After the RI had elapsed, a second fixation point (differently coloured from the first fixation point) was presented to ensure the subjects did not solve the spatial task by holding their bill over the location of the sample square. The choice phase was presented after the second fixation point was pecked. There were three choices (two distracters and the correct sample square) presented for both tasks and so accuracy expected by chance was 0.33. The subject had to choose the square that was in the same location (spatial task) or the same colour (colour task) as the sample square (S1 Fig). A correct choice was rewarded with 5 s of food access and an incorrect choice was followed by a black screen that darkened the chamber for 5 s. Each subject was tested in 1.5 h daily sessions during which subjects completed as many trials as possible (from 20 to over 200 trials, depending on RI length). The illumination level of the white screen on which stimuli were presented was 116.3 lux and the illumination level of the black screen was 2.2 lux measured at the position of the bird in the chamber.

Subjects were trained on a 0-s RI task and had to complete at least 5 sessions and reach a criterion of two sessions with ≤ 10% variation and accuracy of at least 10% above random performance (Training to criterion phase in Table 1). Once criterion at a 0 s RI was reached, the RI was increased progressively every fourth session for 15 sessions (Progressive RI phase in Table 1) with a sequence of RIs as follows: 5, 15, 30, 45 and 60 s. The Progressive RI phase was followed by 14 sessions in which the same RI (except 5 s) occurred in a random sequence within each session (Randomized RI phase). The Progressive RI phase and the first 11 sessions in the Randomized RI phase were considered training sessions prior to the subjects reaching their peak performance before subsequent testing for each task type (spatial and colour) and for each breeding condition (non-breeding and breeding) as performance significantly improved over these sessions (Table 1; S1 Text, S1 Fig, S2 Table, S3 Table). Analyses of the Progressive RI phase were not the focus of this study, but are included in the supporting information. Only the last three sessions in the Randomized RI phase were used as testing sessions to assess the subjects’ peak performance described in the sections “DMTS tasks” and “Comparison between spatial and colour tasks” below. In the section “Transition between the spatial and colour tasks”, we used data from the first three sessions on the colour task (Training to criterion phase in Table 1) and the first three sessions in the Progressive RI phase of the colour task, both in non-breeding condition (described in more detail below; Table 1). Each subject thus had extensive training by the onset of the testing sessions, in order to minimize the effects of order of testing (spatial before colour, and non-breeding before breeding; Table 1). One female subject did not reach criterion for the colour task in non-breeding and breeding conditions, therefore we only include her data for the spatial task in non-breeding and in breeding conditions. Removing this female from the analyses did not change the conclusions of our study. For spatial testing in breeding condition, data were absent for two out of the 8 males because one male died and the other fell ill and could not be used for testing during that period. For colour testing in breeding condition, data was missing from the male that previously died and a female that died during that period. Final sample sizes were as follows: non-breeding spatial—8 females, 8 males; non-breeding colour—7 females, 8 males; breeding spatial—8 females, 6 males; breeding colour—6 females, 7 males.

We extensively trained subjects on the colour task before data collection to ensure they were not using a spatial strategy with the colour task. We determined whether the spatial location of the rewarded square affected the subjects’ choice on the colour test (S1 Text). Finally, we analyzed differences in performance between the spatial and colour tasks.

### Statistical analyses

We analyzed our data with linear mixed models (PROC MIXED in SAS 9.3; SAS Institute, Cary NC) because the dependent variables were continuous, repeated measures, and data were missing at random due to the loss of some subjects during the experiment (see above). We used a compound symmetry covariance structure because the model AIC values were lower than with the default variance components covariance structure (see also [42]). Data were appropriately transformed for analyses (see details below) to produce normally distributed residuals. However, to facilitate interpretation, data in figures are presented as untransformed means ± SE, except for significant interactions for the spatial and colour DMTS tasks, which are presented as transformed least squares means ± SE. We analyzed all two-way interactions as factors in our models. Results were considered significant if *p* ≤ 0.05. Significant effects were further analyzed using Fisher’s LSD post-hoc tests.

#### Androgen concentrations

We used a linear mixed model with sex, breeding condition, and task type (spatial or colour) as fixed factors and subject as a random variable to analyze androgen concentrations. Data were log-transformed for analyses to produce normally-distributed residuals.

#### DMTS tasks

We used a linear mixed model for each task type (spatial and colour) with RI (15 s, 30 s, 45 s, and 60 s), breeding condition (non-breeding and breeding), and sex (female and male) as fixed factors and subject as a random variable to analyze the proportion of correct responses, which were arcsine square root transformed for the analyses to produce normally-distributed residuals [43]. The dependent variable was the peak performance (i.e., the last three sessions) from the Randomized RI phase (Table 1).

#### Comparison between spatial and colour tasks

We used a linear mixed model with task type (spatial and colour) and sex (female and male) as fixed factors and subject as a random variable to analyze the proportion of correct responses in the testing sessions, which were arcsine square root transformed for the analyses to produce normally-distributed residuals. The dependent variable was the peak performance (i.e., the last three sessions) from the Randomized RI phase for the spatial and colour tasks (Table 1).

#### Transition between the spatial and colour tasks

Birds performed the spatial task before the colour task first in non-breeding condition and then again in breeding condition (Table 1). To determine whether birds attempted to use a spatial matching strategy to solve the colour matching task, we measured the distance between the sample and the correct square for each trial during the first three training to criterion sessions on the colour task (RI = 0 s, random performance: 0.5) and during the first three sessions in the Progressive RI phase (RI = 5 s, random performance 0.33) of the colour task in non-breeding condition. Random performance was 0.5 during the first three training to criterion sessions because only one distracter was presented during the choice phase. For all birds, this was the first transition from the spatial to the colour task (Table 1). We used linear mixed models for the first three sessions during the initial training and Progressive RI phases with distance between the sample and chosen stimuli and sex as fixed factors and subject as a random variable to analyze the proportion of correct responses. The proportion of correct responses were log arcsine square root transformed for the initial training phase and arcsine square root transformed for the Progressive RI phase to produce normally-distributed residuals.

## Results

Raw data are available in S1 File.

### Confirmation of breeding condition

**Fig 1:**
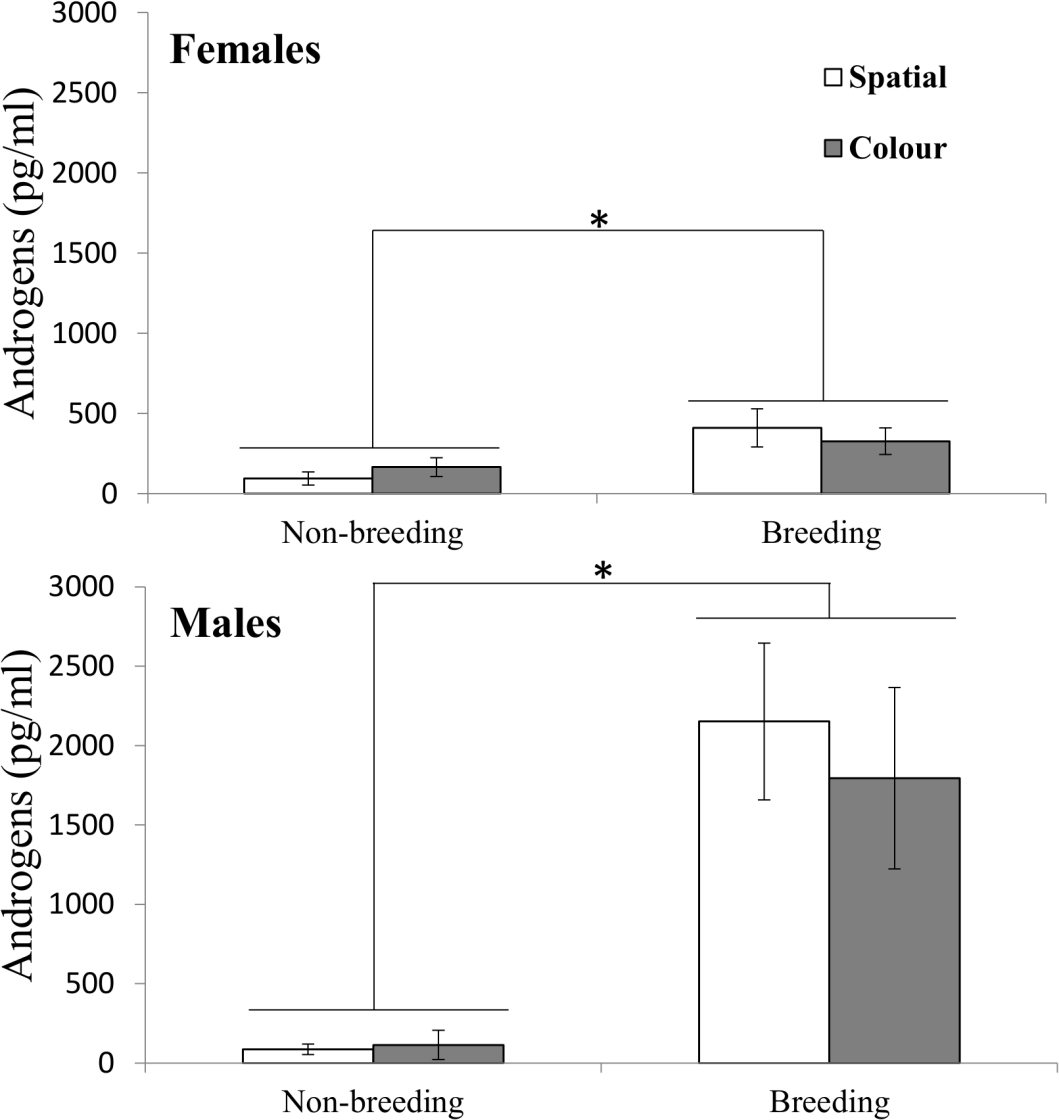
Plasma androgen concentrations in female and male cowbirds between breeding conditions and performance of spatial and colour tasks. Means are presented with ± SE. Asterisks indicate *p* ≤ 0.05.

There was a significant effect of breeding condition, with the highest circulating levels of androgens in breeding condition (F_1,14_ = 120.63, *p <* 0.0001), whereas there was no main effect of sex (F_1,14_ = 2.82, *p =* 0.12), or task type (F_1,14_ = 1.08, *p =* 0.32; Fig 1). There was a significant sex by breeding condition interaction (F_1,14_ = 22.61, *p =* 0.0003), with a greater effect of breeding condition in males (t_14_ = 11.02, *p* < 0.0001) than in females (t_14_ = 4.45, *p* = 0.0005; Fig 1). There was also a significant task type by breeding condition interaction (F_1,14_ = 5.95, *p =* 0.03), with a greater change in androgen concentration between breeding conditions for the spatial task (t_13_ = 9.59, *p* < 0.0001) than for the colour task (t_13_ = 5.98, *p* < 0.0001; Fig 1). There was no significant interaction between sex and task type (F_1,14_ < 0.01, *p =* 0.99).

### Testing

#### Spatial memory

**Fig 2:**
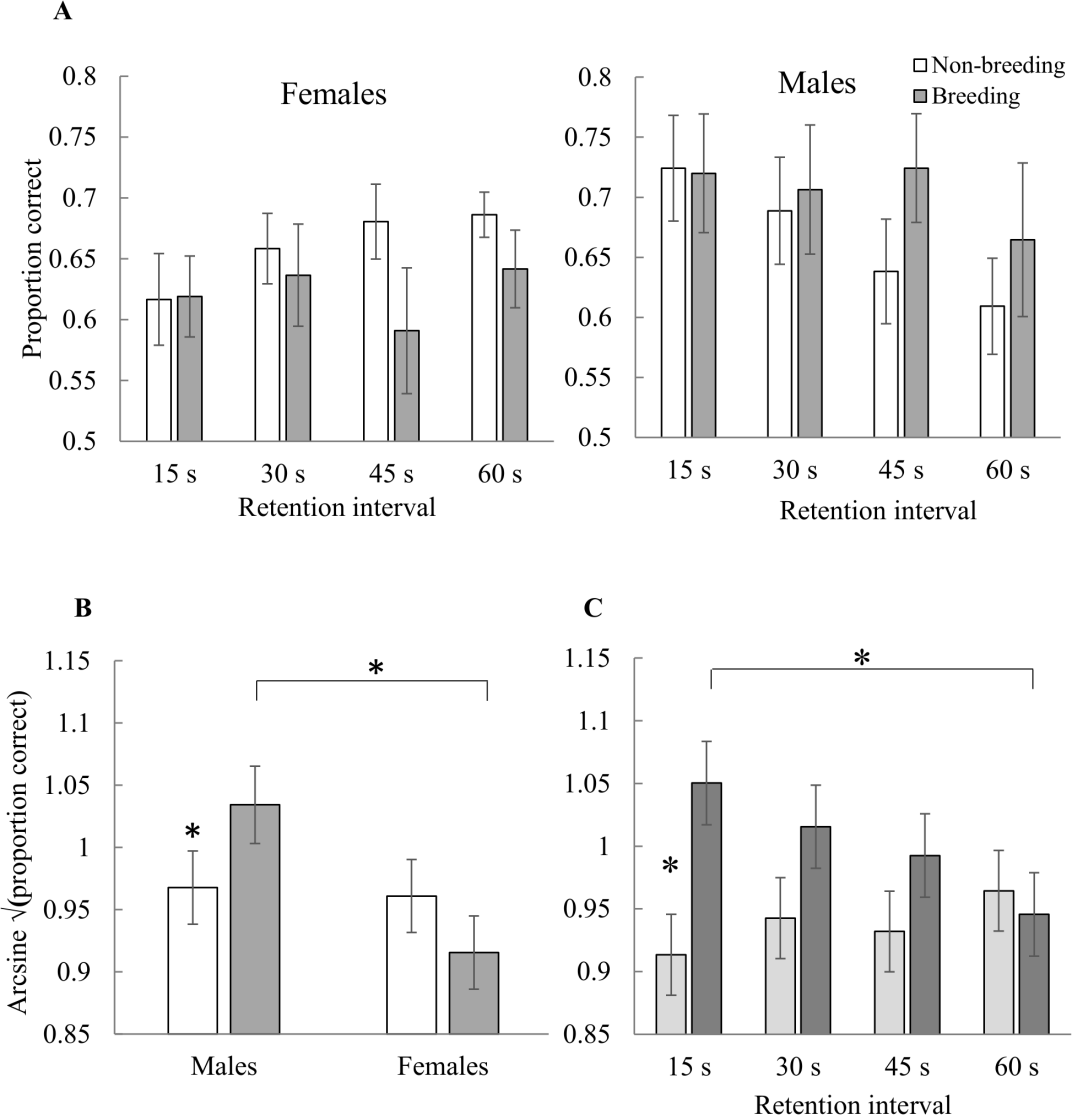
A) Peak performance on the spatial delayed-matching-to-sample touchscreen task. Performance was calculated from the last three sessions of the randomized retention intervals phase. Means of raw data are presented with ± SE. Proportion correct expected by chance equals 0.33. Two males were only tested in non-breeding condition and their performance was weighed more heavily in the raw means than in the linear mixed models, for which least squares means of significant interactions are presented in B and C. B) Summary by sex and breeding condition of data shown in A), showing least squares means ± SE of arcsine square root transformed data. Breeding males were significantly better than non-breeding males, but there was no difference between non-breeding and breeding females. In addition, breeding males performed significantly better than breeding females. C) Summary of data shown in A) by retention interval (RI), showing least squares means ± SE of arcsine square root transformed data. Males performed significantly better than females at the 15s RI. Males performed significantly worse at the 60s RI than at the 15s RI. Females are in light grey and males are in dark grey. Asterisks indicate *p* ≤ 0.05.

There were no main effects of sex, breeding condition, or RI and no significant breeding condition by RI interaction on performance (0.19 ≤ *p* ≤ 1.00; Fig 2A; S1 Text, S2 Table). There was, however, a significant sex by breeding condition interaction (F_1,12_ = 11.71, *p* = 0.005; Fig 2B). Breeding males performed better than breeding females (t_12_ = 2.42, *p =* 0.03; Fig 2B). In addition, males performed better in breeding condition than in non-breeding condition (t_12_ = 2.70, *p =* 0.02) whereas females performed nearly significantly better in non-breeding condition than in breeding condition (t_12_ = 2.11, *p =* 0.06; Fig 2B). There was also a significant sex by RI interaction (F_3,42_ = 4.06, *p* = 0.01), with, males performing better than females at the 15 s RI (t_42_ = 2.57, *p =* 0.01; Fig 2C). Males performed more poorly at the 60s RI compared to the 15 s RI (t_42_ = 3.19, *p =* 0.003), whereas females’ performance remained stable (t_42_ = 1.67, *p =* 0.10; Fig 2C).

#### Colour memory

**Fig 3:**
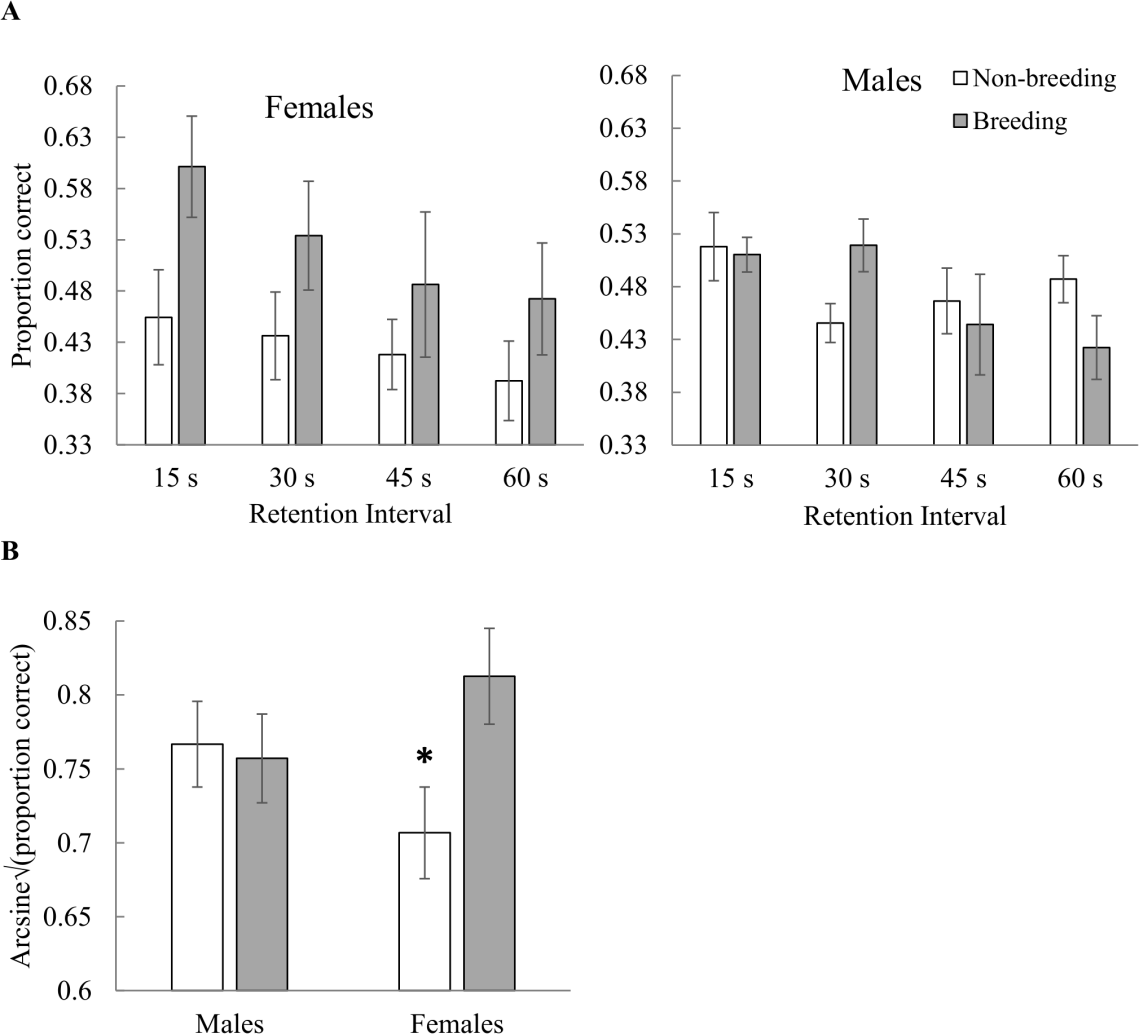
A) Peak performance on the colour delayed-matching-to-sample touchscreen task. Performance was calculated from the last three sessions of the randomized retention intervals phase. Means of raw data are presented with ± SE. Proportion correct expected by chance equals 0.33. One female and one male were only tested in non-breeding condition and these missing points were corrected for in our linear mixed model, for which the only significant interaction is shown in B. B) Summary of the data shown in A) by sex and breeding condition, showing least squares means ± SE of arcsine transformed square root data. Females performed significantly better in breeding than in non-breeding condition, with no effect of breeding condition for males. Asterisks indicate *p* ≤ 0.05.

There was a significant main effect of breeding condition (F_1,11_ = 8.60, *p* = 0.01), however, this effect was driven by females as there was a significant sex by breeding condition interaction (F_1,11_ = 12.41, *p =* 0.005), with females performing better in breeding condition than in non-breeding condition (t_11_ = 4.40, *p =* 0.001) and males’ performance showing no difference between breeding conditions (t_11_ = 0.44, *p* = 0.67; Fig 3). There was also a significant main effect of RI, with performance decreasing as RI increased (F_3,39_ = 5.52, *p =* 0.003; Fig 3A). There was no effect of sex and all other interactions were not significant (0.14 ≤ *p* ≤ 0.96; S1 Text, S3 Table).

#### Comparison between spatial and colour tasks

**Fig 4:**
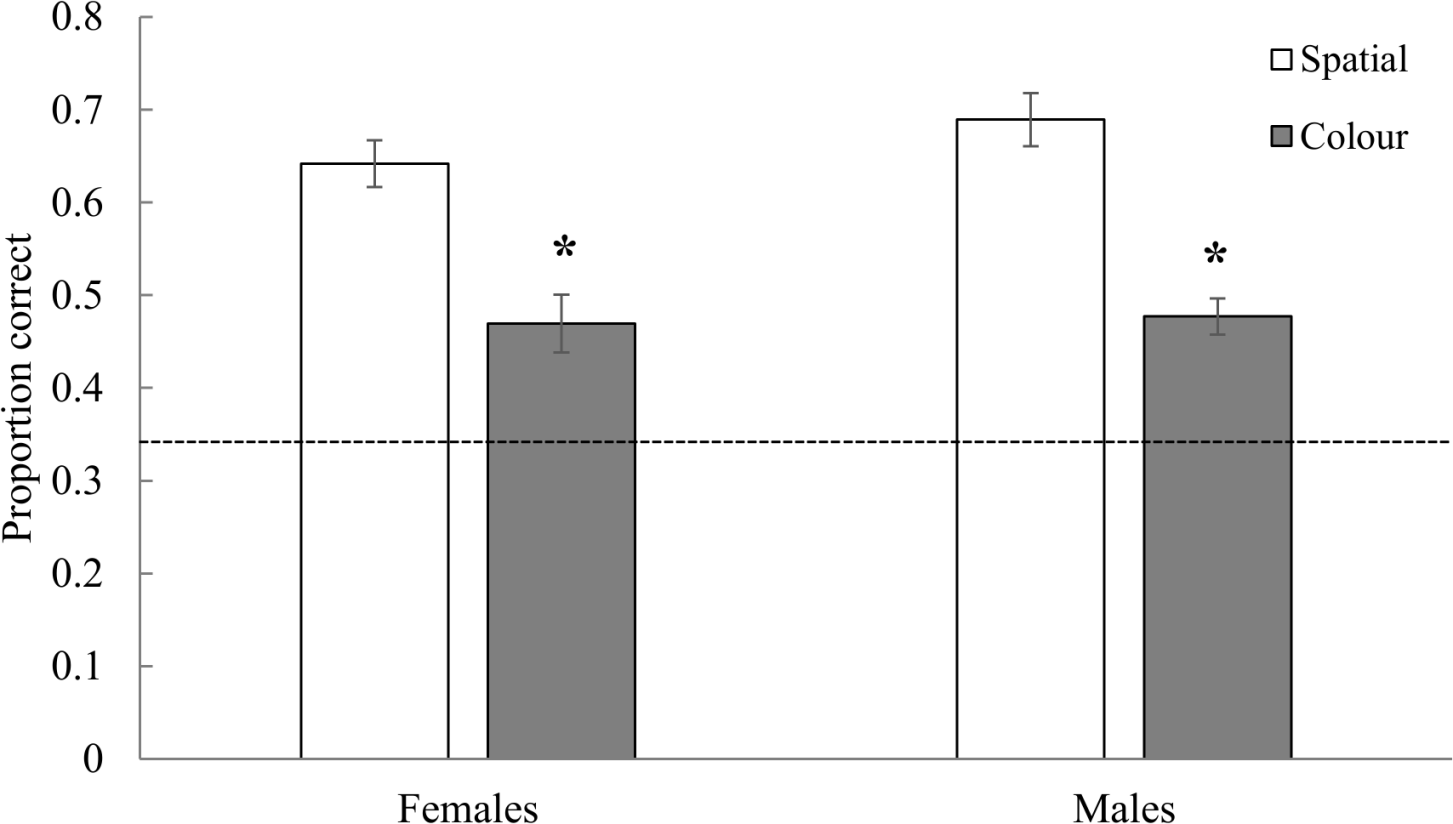
Comparison of performance between the spatial and colour tasks during the testing sessions (see Fig 1). Female and male cowbirds performed significantly better on the spatial task than on the colour task, indicated by asterisks. Performance of both females and males was significantly better than chance, indicated by the dashed line. Means are presented with ± SE. Asterisks indicate *p* ≤ 0.05.

Performance differed significantly between task types (F_1,13_ = 310.20, *p <* 0.0001), with cowbirds performing better on the spatial task than on the colour task (Fig 4). There was no significant main effect of sex (F_1,14_ = 1.22, *p* = 0.29) and no significant sex by task interaction (F_1,13_ = 1.10, *p* = 0.31; Fig 4).

#### Transition between spatial and colour tasks

**Fig 5:**
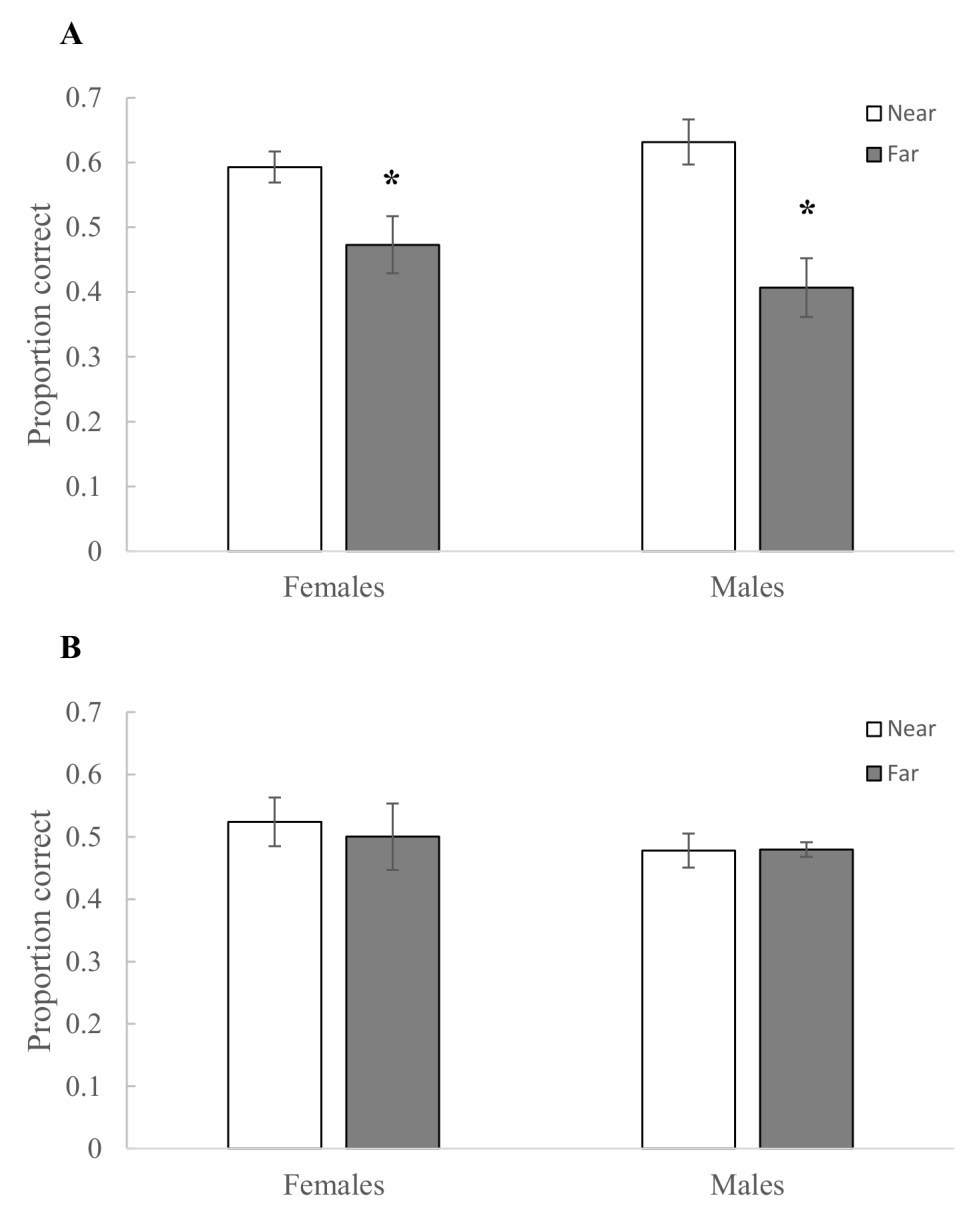
Transition from spatial to colour delayed-matching-to-sample touchscreen tasks. The figure shows the proportion correct in the colour task when the correct match was near or far from the spatial location of the sample. A) In the first three sessions on the colour task (retention interval [RI] = 0 s), both males and females were more likely to make the correct colour choice when the matching stimulus was near the location the sample had occupied. Random performance was 0.5 (dashed line) because there was only one distracter presented during the choice phase. B) After two weeks of testing, when the Progressive RI testing phase began (RI = 5 s), the location of the matching colour stimulus had no effect on performance. Random performance was 0.33 (dashed line) because there were two distracters presented during the choice phase. Means are presented with ± SE. Asterisks indicate a significant difference (*p* ≤ 0.05) between near and far trials.

During the initial transition from the spatial to the colour task (i.e., in non-breeding condition), birds were more likely to respond correctly when the correct colour match was near the spatial location of the sample than when it was further away (F_1,13_ = 43.96, *p* < 0.0001) (Fig 5A). There was no main effect of sex (F_1,13_ = 0.60, *p =* 0.45; Fig 5A), but there was a nearly significant interaction between sex and distance (F_1,13_ = 4.42, *p =* 0.06), with distance affecting males (t_13_ = 6.32, *p <* 0.0001) more than females (t_13_ = 3.13, *p =* 0.008; Fig 5A).

When birds were trained on the Progressive RI colour task in non-breeding condition, following two weeks of training and the birds had reached criterion for the colour task, there was no longer an effect of distance on performance (F_1,13_ = 0.28, *p* = 0.61; Fig 5B). There was no main effect of sex (F_1,13_ = 0.90, *p* = 0.36) and no significant sex by distance interaction (F_1,13_ = 0.36, *p* = 0.56; Fig 5B). Thus, birds initially used a spatial strategy to solve the colour task, but by the beginning of Progressive RI training on the colour task, they no longer did so.

## Discussion

Male cowbirds were more accurate than females on the spatial touchscreen task in two ways: first, males performed better than females at a short RI (15 s) and second, breeding males performed better than breeding females (Fig 2). On the colour task, females performed better in breeding than in non-breeding condition, whereas males’ performance remained stable between breeding and non-breeding conditions (Fig 3). Finally, females and males both performed better on the spatial task than on the colour task (Fig 4).

Order effects could have potentially influenced differences in performance between tasks and between breeding conditions, because cowbirds were tested on the spatial task before the colour task and in non-breeding condition before breeding condition (Table 1). However, we found order effects were negligible for task order because spatial location did not influence performance during the Progressive RI phase of the colour task, several sessions before colour testing began (Fig 5). This lack of order effects was likely due to extensive practice on the colour task before testing began (Table 1). In a similar fashion, birds were trained once again to asymptotic performance in breeding condition before the onset of the Progressive RI phase so that birds began training with increasing RI while at their peak performance at a 0 s RI (Table 1). We would expect birds of both sexes to perform better in breeding condition on both tasks if order effects were the main factors influencing differences between breeding conditions. However, males’ performance remained stable between breeding conditions on the colour task and females performed nearly significantly better in non-breeding condition than in breeding condition on the spatial task (Fig 2). We cannot fully disentangle breeding condition versus non-breeding condition from the passage of time but explanations of the results based on order effects alone seem unlikely.

### Spatial memory

In contrast to the results of the current study, female cowbirds had more accurate spatial memory than males when navigating through a room to find baited food cups [21]. Although the task in that prior study and the touchscreen task described here were both DMTS tasks assessing spatial memory, the two tasks differed in spatial scale (180 cm X 180 cm versus 8.5 X 8 cm) and retention interval (24 h versus 5–60 s). Furthermore, the tasks differed in the response required of the birds; approaching and feeding from a cup versus pecking a symbol on a screen. Thus, whether or not a sex difference in spatial memory in cowbirds is observed depends on the spatial task.

Sex differences in spatial ability can be task-dependent. Males, especially in mammals, perform better than females on a variety of spatial tasks and a consistent feature of their enhanced performance is the use of spatial cues (geometric properties of the environment, distance, and direction) and feature cues while females prefer to use only feature cues [7, 8, 17, 44, 45]. However, recent work in birds showed that preference for spatial cues is not restricted to males but depends on the value of a cue to the solution of the task [23, 24]. Female cowbirds may have had a preference for spatial cues over feature cues on the task in [21], but this preference was reduced or absent in the current task. Selection may have led to flexibility in cue use in cowbirds rather than consistently superior spatial ability by one sex or the other [23, 24]. Finally, females performed equally well, and above chance, across retention intervals while male performance declined significantly with retention interval (Fig 2). Females may be relatively unaffected in general by retention interval. In the wild, females probably remember the locations of potential host nests for at least 24 h and females outperformed males at the 24 h retention interval tested in [21].

Why did male cowbirds outperform females in breeding condition? From a life-history perspective, there could be a trade-off associated with specialization in a particular form of memory. Enhanced cognitive function has metabolic and life-history costs [46, 47, 48, 49]. Improved ability in one type of spatial memory may come at the cost of another type of spatial memory. Female cowbirds did better on an allocentric task in which they moved through their environment and this ability may help females find and relocate host nests [21]. Enhanced performance by female cowbirds in allocentric spatial tasks may come at a cost in the performance of egocentric tasks such as our spatial touchscreen task in the current study. It is also possible that there is differential selection on males—for unknown reasons—for the ability to remember the location of objects in a spatial array in their immediate visual field. Finally, it is possible there is some functional incompatibility [50]—again for reasons that are not known—between different kinds of spatial ability involved in orientation and navigation and remembering the location of objects in an array. In humans, men outperform women on several measures of wayfinding through a wooded area or a large indoor environment [17, 44] but women outperform men on stationary object location memory tasks [18, 51]. In cowbirds, observed sex differences on these different kinds of spatial memory are reversed relative to humans.

A proximate explanation for superior male performance in breeding condition could be their sex steroid hormone levels. Although androgen concentrations increased in both sexes between breeding conditions, males’ concentration increased a great deal more between non-breeding and breeding conditions (Fig 1). Male deer mice show better acquisition of spatial maze performance than females in breeding condition only, when their testosterone levels are highest [12]. In a task similar to ours, exogenous androgens improved performance on a delayed-non-matching-to-sample touchscreen task in great tits [35]. In situ hybridization shows expression of androgen and estrogen receptor genes in the hippocampus of tits and testosterone and estrogen could influence spatial memory by binding to these receptors [35]. The songbird hippocampus expresses high levels of aromatase, resulting in high levels of local estrogen synthesis from testosterone and enhanced spatial memory acquisition and performance [34, 36, 52]. Enhanced performance on the DMTS spatial task by male cowbirds could thus have been caused by elevated testosterone levels.

#### Colour memory

Why might colour memory improve in breeding condition for females, but not for males (Fig 3)? One ultimate explanation could be that improved colour memory is related to mate choice. The plumage of male cowbirds that are nutritionally stressed has a lower brightness, hue, and saturation than the plumage of males that are fed *ad libitum* [53]. Females generally prefer to mate with males exhibiting the brightest ornaments [54] and female colour memory during breeding may contribute to successful mate choice.

Another ultimate explanation may be that females use colour cues to find suitable host nests to parasitize. Although cowbirds are considered host generalists, they do show host selectivity [55, 56]. Cowbirds parasitize preferred hosts first, but switch to less preferred hosts when others are unavailable [56]. Host eggs and host themselves vary in colour [55] and colour could be an important cue in nest selection.

A proximate explanation for better performance by females on the colour task in breeding condition could be that colour vision differs between the sexes and may vary with breeding condition. Female cowbirds have poorer chromatic visual resolution than males [57]. Based on their time of capture, the birds examined by [57] were probably in non-breeding condition. There may, thus, be differences between male and female cowbirds in colour vision and the nature of this difference may vary with breeding condition

### Spatial versus colour memory

Both female and male cowbirds performed better on the spatial task than on the colour task (Fig 4). We showed that birds did not use a spatial strategy to solve the colour task early in the Progressive RI phase, several sessions before colour testing (Fig 5). This result suggests that the effect of order (spatial before colour) was minimized or possibly eliminated with extensive practice on the colour task, although we cannot completely rule out potential order effects.

Better performance on the spatial task relative to the colour task may simply be the result of how spatial and colour memory was tested and not a general difference between spatial and colour memory in cowbirds. The colour task may have been more difficult because of the difference in perceptual space between the correct match and distractors, or the colour samples may have been less memorable. Alternatively, species that have strong demands on spatial memory may perform better on spatial than colour tasks. For example, food-storing black-capped chickadees (*Poecile atricapillus*) performed better on a spatial touchscreen task than on a colour touchscreen task whereas non-food-storing dark-eyed juncos (*Junco hyemalis*) performed equally well on both tasks [58]. Enhanced performance on the spatial task in chickadees was proposed to be related to the chickadees’ reliance on memory for the location of stored food in the wild [58]. Other food-storing species are better than non-food-storers on spatial DMTS touchscreen tasks similar to ours [59]. Free-living cowbirds, both males and females, may rely more heavily on spatial information than on colour information in behaviours resembling our DMTS task, such as foraging for seeds or invertebrates on the ground. It is also possible that sex-specific selection acting on females for enhanced spatial memory has affected males. Many genes are obviously shared between the sexes and this genetic correlation can cause a trait favored in one sex to occur in both [60, 61]. A test of this hypothesis, would be to compare performance of male and female icterids that are not brood parasites on the spatial and colour DMTS tasks and the orientation task used in [21].

### Conclusions

Sex differences in spatial memory in brown-headed cowbirds depend on the task used to assess spatial ability. Females performed better than males in a related study in which birds moved through their testing environment to return to a remembered spatial location. However, male cowbirds performed better than females on a stationary spatial task in the current experiment. This task-dependent sex difference in spatial memory is the reverse of that observed in humans and could be due to several factors such as trade-offs between different forms of spatial memory. Colour memory was found to improve in females from non-breeding condition to breeding condition and may play a role in host nest selection or mate choice. Finally, males and females alike were better on the spatial task than on the colour task, suggesting that memory for spatial information may be more accurate than memory for colour information in both sexes in some contexts in their natural environment.

## Supporting Information

S1 Fig Diagram of spatial and colour delayed-matching-to-sample tasks. Spatial (top) and colour (bottom) delayed-matching-to-sample (DMTS) tasks. Every trial began with a fixation point (first image from left), followed by a sample square (second image from left). The bird had to remember either the location (spatial DMTS) or the colour (colour DMTS) of the sample square. After pecking the sample square, it disappeared and a retention interval (RI) of 5, 15, 30, 45 or 60 s with a blank white screen was displayed. After the RI, a second fixation point (third image from left) was displayed. The bird had to peck the second fixation point to have a choice of three squares displayed (last image). The correct square was either in the same location (spatial DMTS) or the same colour (colour DMTS) as the sample square. A correct choice resulted in 5 s of food access with a blank white screen and an incorrect choice resulted in 5 s without food access with a blank black screen.
(TIFF)

S2 Fig Performance during the Progressive retention interval (RI) phase. Mean performance ± SEM on the Progressive RI spatial (top) and colour (bottom) delayed-matching-to-sample tasks in non-breeding and breeding conditions. The retention interval was progressively increased after three sessions at a given retention interval.
(TIFF)

S1 File Raw data. (XLSX)

S1 Table Colours for the colour task. HTML codes for the colours used in the colour delayed-matching-to-sample task.
(PDF)

S2 Table Detailed statistics for the spatial task. Summary of statistical effects of sex, breeding condition (BC), retention interval (RI) and their interactions during the 15 practice sessions from the Progressive RI phase and the 3 test sessions from the Random RIs phase for the spatial delayed-matching-to-sample task. Data were log-arcsine transformed for the Progressive RI and arcsine transformed for the Randomized RI to produce normally distributed residuals. Significant effects are in bold.
(PDF)

S3 Table Detailed statistics for the colour task. Summary of statistical effects of sex, breeding condition (BC), retention interval (RI) and their interactions during the 15 practice sessions from the Progressive RI phase and the 3 test sessions from the Random RIs phase for the colour delayed-matching-to-sample task. Data were arcsine transformed for the Progressive RI and Randomized RI to produce normally distributed residuals. Significant effects are in bold.
(PDF)

S1 Text Detailed methods and results. (PDF)
