# Supplementary figures and images for: Sex Differences in Spatial Memory in Brown-Headed Cowbirds: Males Outperform Females on a Touchscreen Task
Source: PLoS One. 2015 Jun 17;10(6):e0128302. doi: 10.1371/journal.pone.0128302 (PMC4470821; doi:10.1371/journal.pone.0128302)

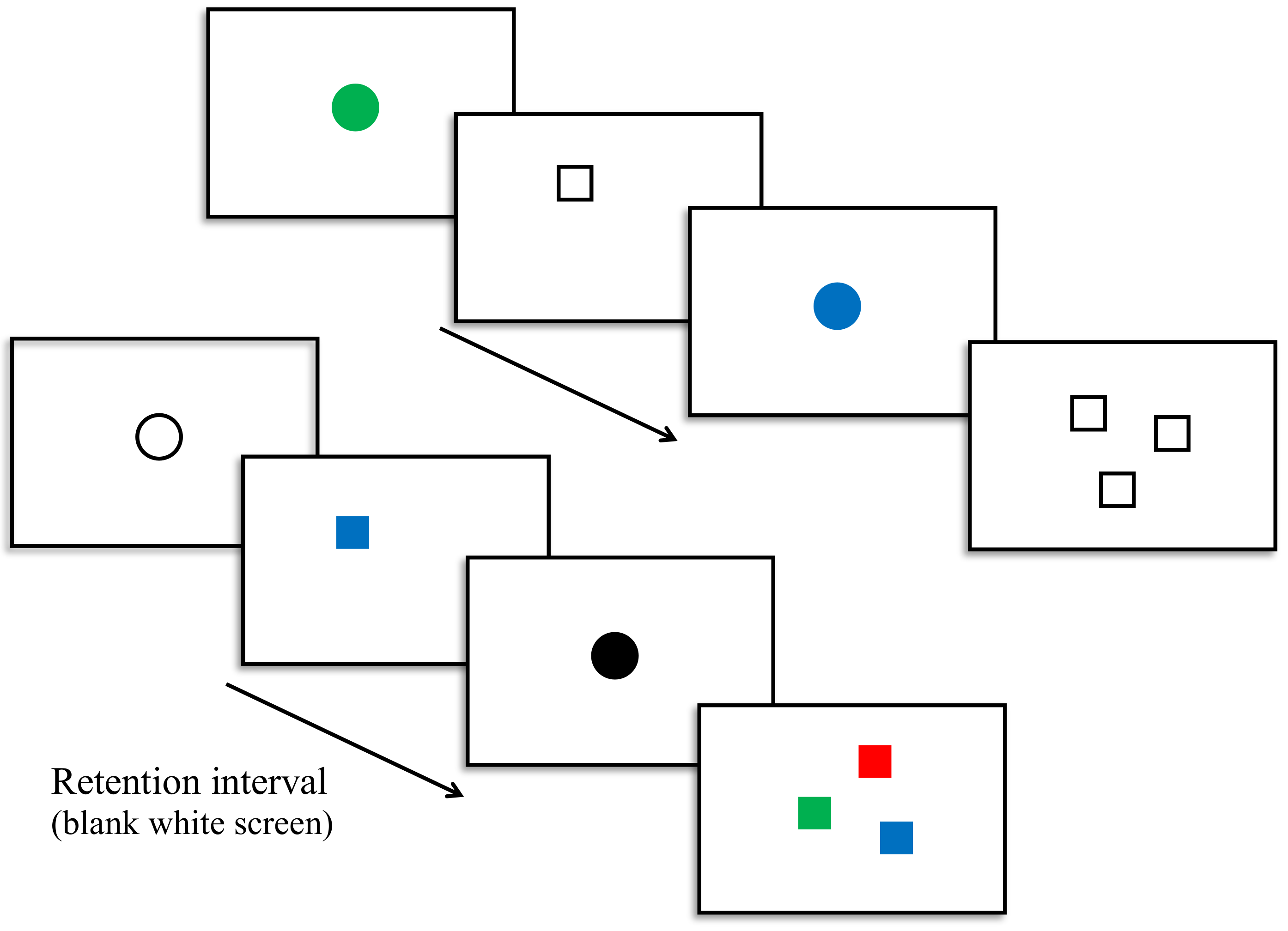

Supplement: S1 Fig — Spatial (top) and colour (bottom) delayed-matching-to-sample (DMTS) tasks. Every trial began with a fixation point (first image from left), followed by a sample square (second image from left). The bird had to remember either the location (spatial DMTS) or the colour (colour DMTS) of the sample square. After pecking the sample square, it disappeared and a retention interval (RI) of 5, 15, 30, 45 or 60 s with a blank white screen was displayed. After the RI, a second fixation point (third image from left) was displayed. The bird had to peck the second fixation point to have a choice of three squares displayed (last image). The correct square was either in the same location (spatial DMTS) or the same colour (colour DMTS) as the sample square. A correct choice resulted in 5 s of food access with a blank white screen and an incorrect choice resulted in 5 s without food access with a blank black screen. (TIFF) [file pone.0128302.s001.tiff]

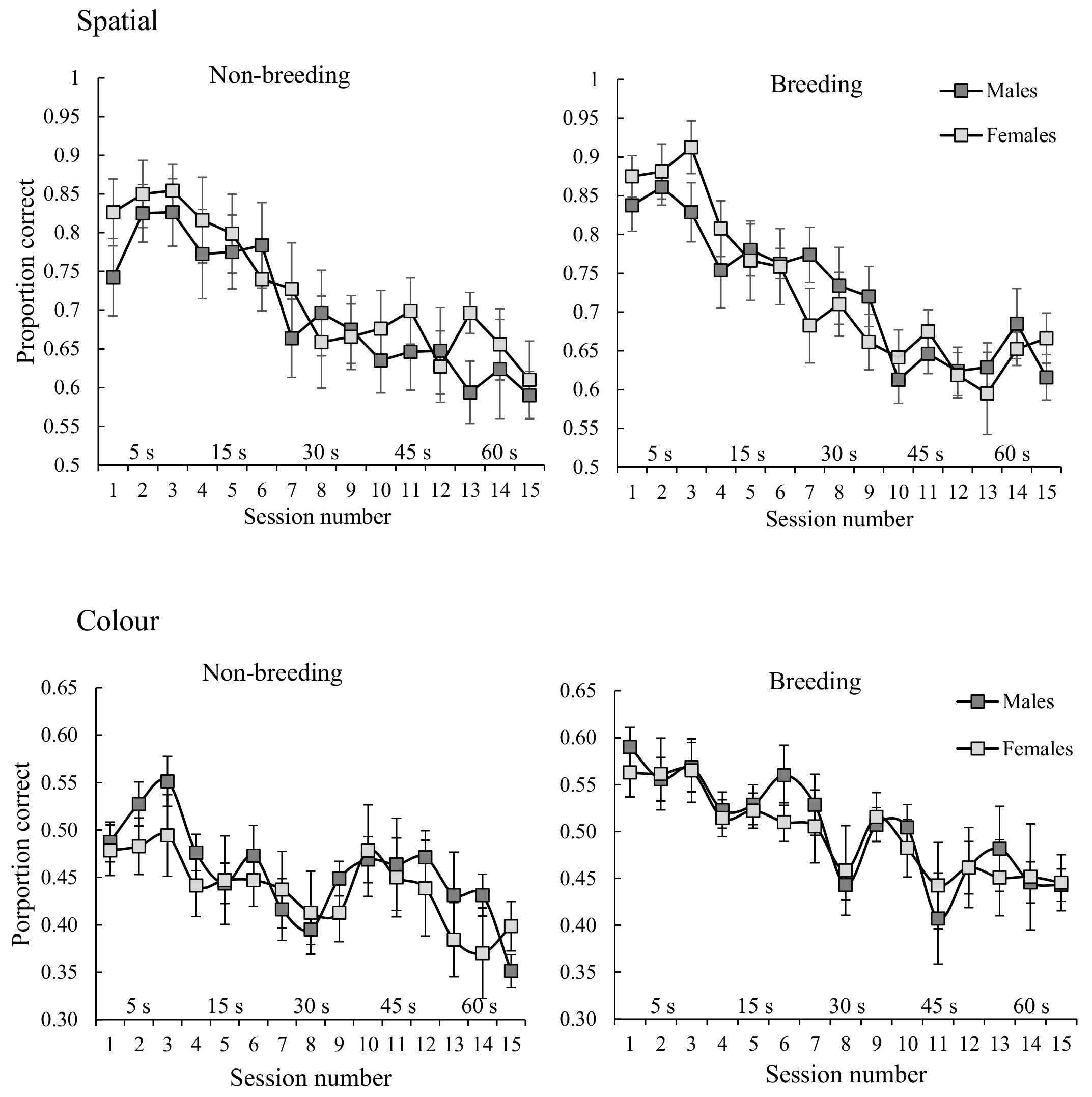

Supplement: S2 Fig — Mean performance ± SEM on the Progressive RI spatial (top) and colour (bottom) delayed-matching-to-sample tasks in non-breeding and breeding conditions. The retention interval was progressively increased after three sessions at a given retention interval. (TIFF) [file pone.0128302.s002.tiff]
