# Supplementary material for: Sex Differences in Spatial Memory in Brown-Headed Cowbirds: Males Outperform Females on a Touchscreen Task
Source: PLoS One. 2015 Jun 17;10(6):e0128302. doi: 10.1371/journal.pone.0128302 (PMC4470821; doi:10.1371/journal.pone.0128302)
Supplement: S1 Table — HTML codes for the colours used in the colour delayed-matching-to-sample task. (PDF) [file pone.0128302.s004.pdf]

Table S1.1. HTML codes for the colours used in the colour DMTS task.

Colour name	HTML code
Green	#55AA2B
Red	#FF0000
Blue	#0000FF
Fuchsia	#FF00FF
Orange	#CC6600
Purple	#800080
Olive	#808000
Dark blue green	#4C7D7E
Brown	#A52A2A
Gold	#EAC117
