# Supplementary material for: Sex Differences in Spatial Memory in Brown-Headed Cowbirds: Males Outperform Females on a Touchscreen Task
Source: PLoS One. 2015 Jun 17;10(6):e0128302. doi: 10.1371/journal.pone.0128302 (PMC4470821; doi:10.1371/journal.pone.0128302)
Supplement: S2 Table — Summary of statistical effects of sex, breeding condition (BC), retention interval (RI) and their interactions during the 15 practice sessions from the Progressive RI phase and the 3 test sessions from the Random RIs phase for the spatial delayed-matching-to-sample task. Data were log-arcsine transformed for the Progressive RI and arcsine transformed for the Randomized RI to produce normally distributed residuals. Significant effects are in bold. (PDF) [file pone.0128302.s005.pdf]

Table S1.2. Summary of statistical effects of sex, breeding condition (BC), retention interval (RI) and their interactions during the 15 practice sessions from the Progressive RI phase and the 3 test sessions from the Random RIs phase for the spatial delayed-matching-to-sample task. Data were log-arcsine transformed for the Progressive RI and arcsine transformed for the Randomized RI to produce normally distributed residuals. Significant effects are in bold.

Factors	*F*	d.f.	*p*
**Spatial**			
Progressive RI – 15 practice sessions			
Sex	0.43	1,14	0.52
BC	1.43	1,14	0.25
RI	66.73	4,56	**<0.0001**
Sex*BC	1.35	1,14	0.26
Sex*RI	1.70	4,56	0.16
BC*RI	1.36	4,60	0.26
Randomized RI – 3 test sessions			
Sex	1.90	1,14	0.19
BC	0.41	1,12	0.53
RI	0.67	3,42	0.57
Sex*BC	11.71	1,12	**0.005**
Sex*RI	4.06	3,42	**0.01**
BC*RI	0.02	3,39	>0.99
