# Supplementary material for: Sex Differences in Spatial Memory in Brown-Headed Cowbirds: Males Outperform Females on a Touchscreen Task
Source: PLoS One. 2015 Jun 17;10(6):e0128302. doi: 10.1371/journal.pone.0128302 (PMC4470821; doi:10.1371/journal.pone.0128302)
Supplement: S3 Table — Summary of statistical effects of sex, breeding condition (BC), retention interval (RI) and their interactions during the 15 practice sessions from the Progressive RI phase and the 3 test sessions from the Random RIs phase for the colour delayed-matching-to-sample task. Data were arcsine transformed for the Progressive RI and Randomized RI to produce normally distributed residuals. Significant effects are in bold. (PDF) [file pone.0128302.s006.pdf]

Table S1.3. Summary of statistical effects of sex, breeding condition (BC), retention interval (RI) and their interactions during the 15 practice sessions from the Progressive RI phase and the 3 test sessions from the Random RIs phase for the colour delayed-matching-to-sample task. Data were arcsine transformed for the Progressive RI and Randomized RI to produce normally distributed residuals. Significant effects are in bold.

Factors	*F*	d.f.	*p*
**Colour**			
Progressive RI – 15 practice sessions			
Sex	0.77	1,13	0.40
BC	44.11	1,12	**<0.0001**
RI	20.21	4,52	**<0.0001**
Sex*BC	0.38	1,12	0.55
Sex*RI	0.48	4,52	0.75
BC*RI	1.25	4,49	0.30
Randomized RI – 3 test sessions			
Sex	<.001	1,13	0.96
BC	8.60	1,11	**0.01**
RI	5.52	3,39	**0.003**
Sex*BC	12.41	1,11	**0.005**
Sex*RI	0.37	3,39	0.78
BC*RI	1.94	3,36	0.14
