# Supplementary material for: Sex Differences in Spatial Memory in Brown-Headed Cowbirds: Males Outperform Females on a Touchscreen Task
Source: PLoS One. 2015 Jun 17;10(6):e0128302. doi: 10.1371/journal.pone.0128302 (PMC4470821; doi:10.1371/journal.pone.0128302)
Supplement: S1 Text — (PDF) [file pone.0128302.s007.pdf]

# Detailed Methods and Results

# Materials

## Shaping and training

Naïve subjects were first placed in the operant chamber with the food hopper in the elevated position until the birds were feeding from the hopper. The hopper was then moved up and down randomly until the birds became habituated to the equipment noise and were feeding from the moving hopper. We then manually shaped the birds to peck a shape on the touchscreen to access the food hopper. We attached a clear tape with seeds over the shape to encourage the birds to peck. It took between one to four 45-min hand-shaping sessions for all the birds to learn to peck a shape to bring the food hopper to the elevated position. Finally, we trained birds to peck progressively longer sequences of shapes until the full sequence for the task was reached.

## Spatial and colour delayed-match-to-sample (DMTS) tasks

A fixation point was displayed until the bird initiated a trial by pecking it, after which this fixation point disappeared and a sample square was presented for a maximum of 90 seconds (S1 Fig.). As soon as the bird pecked the sample square (usually less than 90 seconds), it disappeared and a retention interval (RI) of 5, 15, 30, 45, or 60 s with a blank white screen was displayed. After the RI, a second fixation point was presented for up to 5 s. Once pecked, the second fixation point disappeared and was replaced with a choice of three squares appearing for up to 10 s. If the sample square, the second fixation or a choice was not pecked within the allocated time, the trial was considered

abandoned, a white screen was presented for 5 s and the first fixation point was displayed again to initiate a new trial.

All stimuli were presented within an 85 × 80 mm rectangle on the 340 × 270 mm screen. Fixation points were circles with 30 mm diameter and centred on the screen. The sample and choice (match and distractor stimuli) squares were 25 × 25 mm. Sample squares could appear anywhere around the fixation point, with a consistent distance of 30 mm between the middle of the fixation point and the middle of the sample square. The matching and distractor stimuli appeared 55 mm equidistant from each other rotated randomly around the fixation point and 35 or 65 mm from the sample square for the colour task (see section below for more details). Apart from this constraint, matching and distractor squares could appear at random anywhere around the fixation point except on the spatial task in which one stimulus always occupied the same location as the sample stimulus. The match and distractor stimuli could be no closer to each other than to the comparison stimuli. Colour stimuli for the colour DMTS task could have one of 10 colours. Colours were drawn from HTML codes (S1 Table).

## Transition between spatial and colour DMTS tasks

The squares used in the spatial DMTS task were always white with a black outline and so cowbirds could not use colour to solve the spatial DMTS task. However, cowbirds could have persisted in using spatial location of the sample during the colour DMTS task so we ensured the cowbirds were sufficiently trained on the colour DMTS task before colour testing began. To determine whether location affected the cowbirds' choice during colour testing, we recorded the distance between the location of the sample square presented immediately after fixation point one and the location of the correct matching square during the choice phase. We then determined whether distance influenced the probability of making a correct choice. The correct square was never presented in the same location as the sample

square for the colour task. The distance between the sample and correct squares could either be “near” or “far” because the three choice squares were always equidistant from each other and centered around the fixation point, but shifted either 60º (35 mm; “near”) or 120º (65 mm; “far”) around the fixation point from the original location of the sample square (S1 Fig.). With this information, it was possible to measure the influence of distance on the probability of making a correct choice.

## Breeding condition manipulation and measurement

### Photoperiod manipulation

Subjects were exposed to varying photoperiods at different points throughout the study to induce photosensitive and photorefractory states (Dawson et al. 2000). Subjects in non-breeding condition had been housed with long photophases (16 h: 8 h light:dark [L:D] cycle) for several months, had moulted before testing began, and were thus in a non-reproductive photorefractory state (Dawson et al. 2000). The long photophase was maintained during the entire non-breeding testing period. We then switched birds to a short photophase (8 h L: 16 D) for 60 d to induce photosensitivity. During this period, we did not test birds and they were fed ad libitum. Next, we photostimulated birds with a moderately long photophase (14.5 h L: 9.5 h D) to bring them into breeding condition. Training to criterion began a week later.  We confirmed birds were in non-breeding and breeding condition when photorefractory and photostimulated, respectively, by assaying blood samples from test subjects, noting the frequency of song in the housing room, and examining the gonads of other cowbirds housed in the same room.

### Song recording and laparotomies

We recorded song frequency in the cowbird housing room from 10:00 to 13:00 EDT in the middle of testing in non-breeding condition, three times during breeding and once after testing. Sentinel

cowbirds (birds housed in the same room, but not used in the study) were laparotomized during non-breeding and breeding condition testing. Birds were anesthetised with isoflurane and a small incision was made on the left flank to allow gonad measurement.

# Results

## Confirmation of breeding condition

### Song frequency

Song frequency in non-breeding condition (147 songs per hour) was about a tenth of the frequency recorded in breeding condition (1224, 1098, and 969 songs per hour). After the DMTS testing was completed and the birds moulted, song frequency decreased to a non-breeding level (72 songs per hour).

### Laparotomies

We performed laparotomies on sentinel birds (i.e., birds not used in the DMTS tasks) during non-breeding and breeding conditions to record changes in gonadal development. We measured the testes of three males twice in non-breeding condition (range 1.5 – 2 mm) and two males twice in breeding condition (range 3 – 4 mm), confirming testicular development. We did not monitor changes in ovary morphology via laparotomy. However, near the end of testing in breeding condition three females in the colony laid eggs. One bird laid a single egg, another laid three eggs and one laid one egg per day for 14 consecutive days, confirming that females underwent gonadal development similar to males.

## Acquisition: Progressive RI

### Spatial memory

There was a significant main effect of RI, with performance decreasing as RI increased, whereas sex, breeding condition, and all interactions were not statistically (S2 Table, S2 Fig.).

### Colour memory

There were significant main effects of RI, with performance decreasing as RI increased, and breeding condition, with subjects performing better in breeding condition than in non-breeding condition (S3 Table, S2 Fig.). Sex and all interactions were not significant (S3 Table, S2 Fig.).

# References

Dawson A, King VM, Bentley GE, Ball GF. (2000). Photoperiodic control of seasonality in birds. Journal of Biological Rhythms, 16:365-380.
